# Influenza A H5N1 Replication Sites in Humans

**DOI:** 10.3201/eid1107.041313

**Published:** 2005-07

**Authors:** Mongkol Uiprasertkul, Pilaipan Puthavathana, Kantima Sangsiriwut, Phisanu Pooruk, Kanittar Srisook, Malik Peiris, John M. Nicholls, Kulkanya Chokephaibulkit, Nirun Vanprapar, Prasert Auewarakul

**Affiliations:** * Mahidol University, Bangkok, Thailand;; † University of Hong Kong, Hong Kong Special Administrative Region, People's Republic of China

**Keywords:** Avian influenza A virus, autopsy, pathogenesis, lung, intestine, TNF

## Abstract

Tissue tropism and pathogenesis of influenza A virus subtype H5N1 disease in humans is not well defined. In mammalian experimental models, H5N1 influenza is a disseminated disease. However, limited previous data from human autopsies have not shown evidence of virus dissemination beyond the lung. We investigated a patient with fatal H5N1 influenza. Viral RNA was detected by reverse transcription–polymerase chain reaction in lung, intestine, and spleen tissues, but positive-stranded viral RNA indicating virus replication was confined to the lung and intestine. Viral antigen was detected in pneumocytes by immunohistochemical tests. Tumor necrosis factor-α mRNA was seen in lung tissue. In contrast to disseminated infection documented in other mammals and birds, H5N1 viral replication in humans may be restricted to the lung and intestine, and the major site of H5N1 viral replication in the lung is the pneumocyte.

Highly pathogenic avian influenza virus H5N1 is the first avian influenza virus that was documented to cause respiratory disease and death in humans (1–3). In 2004, it caused widespread disease in poultry in Asia (4) and led to human disease in Thailand and Vietnam, with reported fatality rates of 66% and 80%, respectively (5,6). With the emergence of a second wave of disease outbreaks in poultry in Thailand, Vietnam, and Indonesia, this disease poses a global threat to human health (4). Additional human cases have been reported since August 2004. The high pathogenicity of this virus in avian species is associated with readily cleavable hemagglutinin (HA), but other amino acid residues in HA and neuraminidase have been recently reported to be involved in avian pathogenicity (7). In mice, some H5N1 virus strains cause a disseminated infection and death, and this phenotype was associated with specific amino acid substitutions in PB2 and the multibasic cleavage site in HA (8). Natural infection of felines with H5N1 viruses also resulted in disseminated infection (9). However, the pathogenesis of H5N1 disease in humans is more obscure. Despite severe and generalized clinical manifestations, the result of multiple organ dysfunction, previous limited autopsy data failed to show evidence of viral replication beyond the respiratory tract (10,11). The tissue tropism of the virus in humans has also not been clearly established by immunohistochemical analyses (10,11). The absence of detectable viral antigen–positive cells in previous reports may relate to the fact that the patients died during the late phase of the disease after intensive treatment with antiviral drugs. In this report, we investigated a case of fatal H5N1 disease in a child for tissue tropism caused by the virus in the lungs and other organs.

## Methods

### Patient and Virologic Diagnosis

Detailed clinical description of the patient is reported elsewhere (12). The patient was a 6-year-old boy who had a progressive viral pneumonia that led to acute respiratory distress syndrome and death 17 days after onset of illness. He was initially treated with multiple broad-spectrum antimicrobial agents. Virologic diagnosis of H5N1 infection was made on day 7 of illness. After oseltamivir became available in Thailand, he was treated on day 15 of his illness with this agent until he died. He was also treated with methylprednisolone on day 15 until death and with granulocyte colony-stimulating factor for leukopenia from day 5 to day 10 of illness.

Virologic diagnosis was made by antigen detection, viral culture, and reverse transcription–polymerase chain reaction (RT-PCR) on a nasopharyngeal wash specimen as described (12) and was confirmed by seroconversion of neutralizing antibody against H5N1 virus. The virus was identified as avian influenza virus (H5N1) by whole genome sequencing. The virus was an avian virus with no evidence of genetic reassortment with human influenza viruses. Phylogenetic analysis showed that the viral genomic sequence formed a distinct cluster with other H5N1 viruses isolated from humans and poultry in Thailand and Vietnam, but it was still related to the previously described H5N1 viruses circulating in southern China. As with other viruses isolated from poultry in Vietnam, Thailand, and Indonesia, this virus was also a genotype Z virus (4).

### Pathologic Examination

Autopsy was carried out by standard techniques, and precautions were taken to minimize risk of transmission of infection. The tissue obtained was prepared for routine histologic analysis, and a portion was stored at –70°C for further study. For RT-PCR, fresh unfixed specimens were minced into small pieces in lysis buffer of an RNA extraction kit (RNA Wizard, Ambion, Austin TX, USA). Total RNA was then extracted according to the manufacturer's protocol. RNA was also extracted from paraffin-embedded tissues by sequential extraction with TriZol reagent (Invitrogen, Carlsbad, CA, USA) and the RNAEasy kit (Qiagen, Valencia, CA, USA) after digestion with proteinase K. RT-PCR for H5 was then conducted on extracted RNA by using One Step RT-PCR kit (Qiagen) with the H5 specific primer pairs H5F (5´-ACTCCAATGGGGGCGATAAAC-3´) and H5R (5´-CAACGGCCTCAAACTGAGTGT-3´) (13). An RT-PCR for glyceraldehyde-3-phosphate dehydrogenase (GAPDH) mRNA was done in parallel to control for the amount and quality of RNA as described (14). Strand-specific RT-PCR was carried out by a method similar to RT-PCR for viral RNA detection, except that only 1 primer was added at the reverse transcription step.

For immunohistochemical analysis, sections were deparaffinized and rehydrated. Antigenic site retrieval was accomplished by heating each slide in a microwave oven at 700 W for 15 min in 0.05 mol/L citric acid buffer, pH 6.0, and cooling for 20 min at room temperature. Endogenous peroxidase activity was blocked by incubating the slides in 0.3% H_2_O_2_ for 30 min at room temperature. Sections were incubated with 20% normal goat serum (Dako, Glostrup, Denmark) for 20 min at room temperature and then with an anti-influenza A nucleoprotein monoclonal antibody at a 1:100 dilution (B.V. European Veterinary Laboratory, Woerden, the Netherlands) for 1 h at room temperature. Slides were rinsed 3 times in 0.05 mol/L Tris-buffer, pH 7.6, 0.1% Tween 20 and incubated with horseradish peroxidase–conjugated goat anti-mouse immunoglobulin at a 1:400 dilution (Dako) for 30 min at room temperature. The slides were washed as above, developed with diaminobenzidine (Dako), and counterstained with hematoxylin. Some slides of lung tissue were double-stained with a monoclonal antibody (1:50 dilution) against surfactant (Dako).

### Cytokine Expression

Tumor necrosis factor-α (TNF-α), interferon- (IFN-γ), and interleukin-6 (IL-6) mRNA were detected in the extracted RNA by an RT-PCR with previously described primer pairs (15–17). Plasma levels of TNF-α and IFN-γ were measured by enzyme-linked immunosorbent assay (Pierce Endogen, Rockford, IL, USA) and compared with samples from 3 H3 influenza–infected patients and 5 healthy persons.

## Results

**Figure 1:**
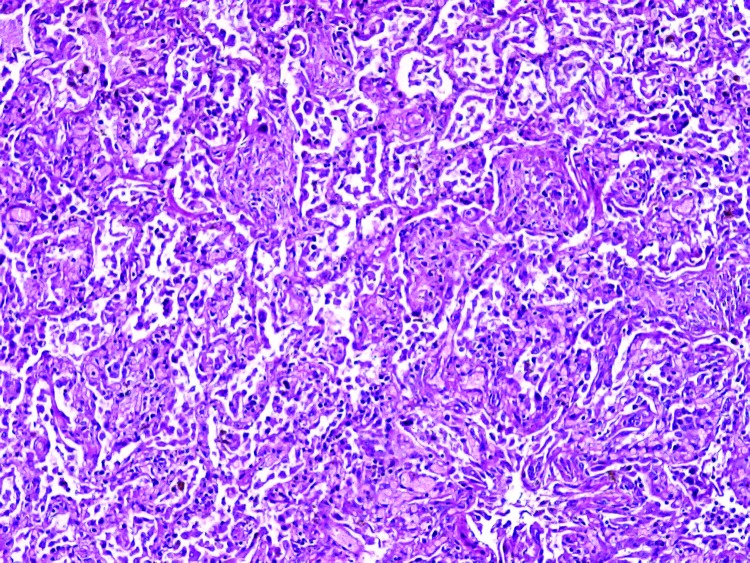
Microscopic shape of the lung showing proliferative phase of diffuse alveolar damage and interstitial pneumonia with reactive hyperplasia of pneumocytes (magnification x100).

The autopsy showed proliferative phase of diffuse alveolar damage, interstitial pneumonia, focal hemorrhage, and bronchiolitis. The pneumocytes showed reactive hyperplasia without virus-associated cytopathic changes (Figure 1). Superimposed infection by fungus, morphologically consistent with aspergillosis, was seen in some areas of the lung. The lymph nodes, spleen, and bone marrow showed slight histiocytic hyperplasia. No evidence of hemophagocytic activity was seen. The liver had mild fatty changes, activated Kupffer cells, and slight lymphoid infiltration in the portal areas. The brain was edematous, and small foci of necrosis were found. Intestines, kidneys, heart, and other organs showed no remarkable changes.

**Figure 2:**
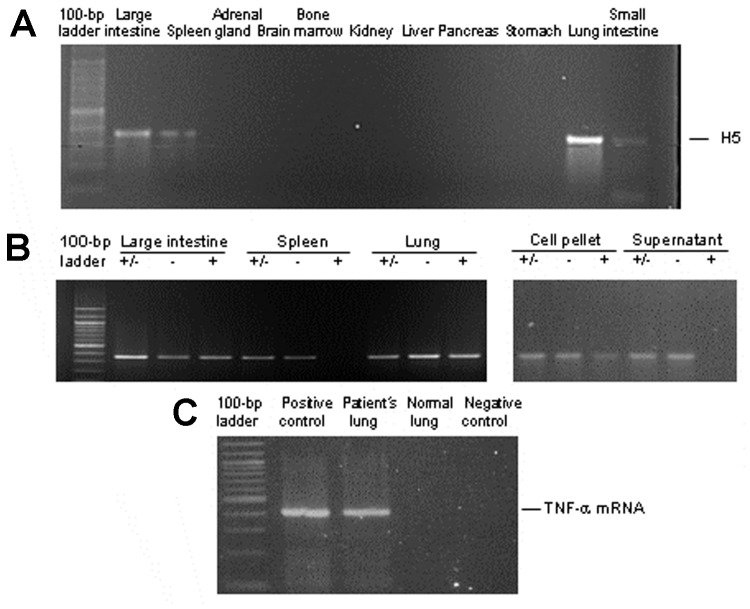
A) Detection of H5 influenza viral RNA in lungs, intestines, and spleen by reverse transcription-polymerase chain reaction (RT-PCR). B) Strand-specific RT-PCR detected positive-stranded viral RNA only in lungs and intestines but not in spleen. +/–, total RNA; –, negative-stranded RNA; +, positive-stranded RNA. RT-PCR products of an infected cell culture pellet and supernatant are shown as a control for proper amplification of the specific strands (lower panel). C) Tumor necrosis factor-α (TNF-α) mRNA was detected by RT-PCR only in lung tissue of the patient but not in lung tissue from a healthy control.

H5-specific RNA was detected in the lung, spleen, and small and large intestines by RT-PCR (Figure 2A). Control reactions without the reverse transcription step were negative, confirming that the PCR amplicon was not contaminated. The successful extractions of RNA from all organs were confirmed by the amplification of GAPDH mRNA (data not shown). We also tested whether the RNA was genomic RNA from virion or replicating RNA and mRNA from productively infected cells. To determine this, we conducted strand-specific RT-PCRs. Positive- and negative-stranded viral RNA was found in the lung, small intestines, and large intestines, but only negative-stranded RNA was detected in the spleen (Figure 2B). Because of the absence of positive-stranded RNA, which would serve as mRNA and the template for genome replication, we concluded that viral replication was absent or very low in the spleen and that the viral RNA detected in the spleen was probably nonreplicating virion RNA. No evidence of viral RNA was seen in the adrenal glands, brain, bone marrow, kidneys, liver, or pancreas. Results of the RT-PCR for viral RNA in plasma were also negative.

**Figure 3:**
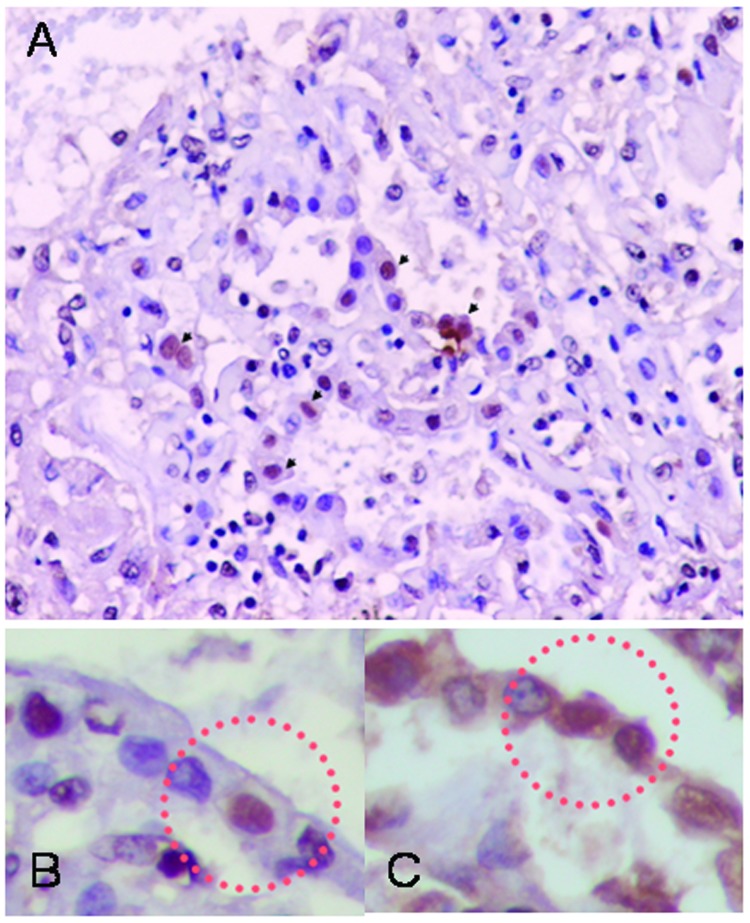
Immunohistochemical analysis showing influenza A antigen-specific staining in nuclei of cells lining the alveoli (A). To identify the cell type, slides from consecutive sections were stained with anti-influenza A antibody (B) and double-stained with antiinfluenza A and antisurfactant antibodies (C). The sections were mapped, and the same area in each section was examined. Viral antigen-positive cells were stained both intranuclearly with antiinfluenza antibody and intracytoplasmically with antisurfactant antibody, indicating that the viral antigen-positive cells were type II pneumocytes. Viral antigen-positive cell are marked by circles (magnification x400).

Immunohistochemical analysis detected influenza A virus antigen-positive cells in lung tissue. The staining was localized in nuclei of alveoli-lining cells. Positive cells were found in 4 of 9 blocks of lung tissue. The shape and location of the antigen-positive cells indicated that they were type II pneumocytes. To confirm this, we used surfactant as a marker of type II pneumocyte (18). We double-stained slides from adjacent cuts with anti-influenza A and anti-surfactant monoclonal antibodies and showed that all influenza virus antigen–positive cells with nuclear staining showed intracytoplasmic staining of surfactant (Figure 3). Slides stained only with antibodies to surfactant showed intracytoplasmic, not intranuclear, staining. This finding confirmed that viral antigen–positive cells were type II pneumocytes. Although viral mRNA was present in the intestines, viral antigen was not detected in 4 blocks of tissue from the small and large intestines. In accordance with the absence of viral mRNA in other organs, viral antigen was not detected in those tissues. We also tested 2 blocks of tissue from the trachea. We did not detect any positive staining in columnar epithelium, which is the usual target for influenza virus infection in humans (19), which suggests that the virus targeted primarily lung tissue and not airway epithelium. Similarly, we did not find viral antigen in bronchiolar epithelium in the lung sections. Columnar epithelium in both the trachea and bronchiole was intact, thus providing adequate columnar epithelial cells for evaluation. The lack of pathologic changes is consistent with the absence of viral infection in these tissues.

The high pathogenicity of the H5N1 avian influenza virus has been proposed to be caused by induction of proinflammatory cytokines (20). Cytokine dysregulation could be the major cause of tissue damage in humans, especially in organs in which productive infection does not take place and cell damage cannot be accounted for by cytolytic viral infection. To investigate this aspect of viral pathogenesis, we tested for the presence of cytokine mRNA in tissues from various organs. We detected TNF-α mRNA in lung tissue, but not in other organs (intestines, stomach, spleen, brain, bone marrow, kidneys, liver, and pancreas) of this patient, or in lung tissue of patients who died of other causes (Figure 2C). We did not find any increase in levels of IFN-α, IFN-γ, and IL-6 mRNA in organs of this patient when compared with control tissues from healthy persons.

In accordance with previous reports showing the increased levels of serum cytokines, we found high levels of interferon-induced protein 10 in serum samples collected on day 5 (37,000 pg/mL) and day 10 (4,300 pg/mL) of illness. These levels are comparable to those reported in H5N1-infected cases (10). However, we could not detect any significant levels of TNF-α and IFN-γ in these samples.

## Discussion

Detailed autopsy data on patients with H5N1 disease are limited, and our data provide an insight into the pathogenesis of H5N1 virus in humans. We provide evidence that H5N1 viral replication is not confined to the respiratory tract but may also occur in the gastrointestinal tract. However, a fecal sample was not available for detection of virus. Although viral RNA was detected in the spleen, no evidence of viral replication was seen in this organ. The patient was treated with an antiviral agent for 2 days before death, which could have lowered the level of viral replication in the examined tissues. However, we still found viral mRNA in lungs and intestines, indicating that the viral replication was still ongoing. Viral replication in lungs and intestines was greater than in other sites. Our data agree with previous reports of human cases and cases in experimentally infected macaques, which also suggest that H5N1 influenza virus replication takes place predominantly in the lungs (10,11,21). We also show that type II pneumocytes, not columnar tracheal epithelial cells, are the major site of H5N1 viral replication in humans. Type II pneumocytes are surfactant-producing, alveolar epithelial cells and progenitor cells of both type I and type II cells. This cell type has been shown to contain sialic acid in newborn human lung (22). Whether the availability of the receptor alone determined the site of H5N1 infection needs further investigation.

Infection of the gastrointestinal tract by avian influenza virus, including H5N1, is common in avian species (23,24). However, involvement of the gastrointestinal tract in H1 and H3 influenza infection is rare in humans (25). A patient with H5N1 influenza virus infection was reported to have diarrhea as the initial symptom, which raises the question of whether the gastrointestinal tract may is another site of viral replication and shedding, similar to its function in avian species (26). In another recent report of a patient with a fatal H5N1 infection and severe diarrhea and encephalitis in Vietnam, the virus was found in a rectal swab (27). Our data confirm that H5N1 influenza virus replication can occur in the gastrointestinal tract even in the absence of diarrhea. However, we do not know the extent of viral shedding in stool in this patient. The absence of pathologic changes in the intestine, despite the viral replication, is intriguing.

The absence of viral antigen in the trachea indicated that the upper airway is probably not an active site of the viral replication. This finding is in marked contrast to the circumstances with human influenza, in which the upper respiratory tract and the tracheal and bronchial epithelium are primarily targeted (19). The predilection of H5N1 influenza virus for the lower airways may explain why detecting virus in upper airway specimens for diagnosis of H5N1 infection in humans is difficult (1). This finding also implies that specimens from the lower respiratory tract, such as sputum or bronchoalveolar lavage, would have a higher sensitivity for viral detection than an upper respiratory specimen, such as nasopharyngeal aspirates or throat swab specimens. Our data showing the absence of viral antigen in columnar epithelial cells contrast with a recently published report that H5N1 viral replication took place selectively in ciliated bronchial epithelial cells in an in vitro culture model (28). Whether this result was due to properties of specific viral strains or a difference attributable to the in vitro model needs further clarification.

In contrast to previous reports (10,11), we did not find prominent hemophagocytosis in any of the organs. The presence of hemophagocytosis in these reports supports the cytokine dysregulation model of pathogenesis. Whether the young age of our patient or prior treatment with immunosuppressive corticosteroids affected this manifestation in this patient is unclear.

TNF-α mRNA was detectable in the lungs but not in other tissues. This finding is in agreement with previous observations that H5N1 viruses isolated from human disease hyperinduce production of cytokines, most prominently TNF-α, in cultured human macrophages in vitro (20,29). The simultaneous presence of viral mRNA and cytokine mRNA in the same organ suggests a direct induction of cytokine in productively infected cells. In accordance with this finding, we also found that the viral isolate from this patient induced a high level of TNF-α production from primary human macrophages, which is comparable to the previously described strains (M. Peiris, unpub. data). However, we could not rule out the possibility that the superimposed fungal infection might have played a role in the induction of TNF-α in this patient. The hemagglutinin of the 1918 pandemic H1N1 influenza virus also appears to hyperinduce production of cytokines and chemokines in a mouse model of disease (30).

In conclusion, we have documented that H5N1 disease in humans is one in which viral replication is restricted to the respiratory and gastrointestinal tracts. The multiorgan dysfunction observed in human H5N1 disease, despite the apparent confinement of infection to the lungs, has remained an enigma. The hypothesis that cytokine dysregulation may contribute to the pathogenesis of severe H5N1 disease (20) remains a possibility. An understanding of the pathogenesis of human H5N1 disease is important in preparing for a pandemic.
